# Temperature Behavior of Aqueous Solutions of Poly(2-Oxazoline) Homopolymer and Block Copolymers Investigated by NMR Spectroscopy and Dynamic Light Scattering

**DOI:** 10.3390/polym12091879

**Published:** 2020-08-20

**Authors:** Rafał Konefał, Peter Černoch, Magdalena Konefał, Jiří Spěváček

**Affiliations:** Institute of Macromolecular Chemistry CAS, Heyrovského nám. 2, 162 06 Prague 6, Czech Republic; cernoch@imc.cas.cz (P.Č.); magdalenakonefal@imc.cas.cz (M.K.)

**Keywords:** thermoresponsive polymer, poly(2-ethyl-2-oxazoline), poly(2-isopropyl-2-oxazoline), block copolymers, LCST, DLS, NMR, aqueous solution, NOESY, spin–spin relaxation time

## Abstract

^1^H NMR methods in combination with dynamic light scattering were applied to study temperature behavior of poly(2-isopropyl-2-oxazoline) (PIPOx) homopolymer as well as PIPOx-*b*-poly(2-methyl-2-oxazoline) (PMeOx) and poly(2-ethyl-2-oxazoline) (PEtOx)-*b*-PMeOx diblock copolymers in aqueous solutions. ^1^H NMR spectra showed a different way of phase transition for the main and side chains in PIPOx-based solutions. Additionally, the phase transition is irreversible for PIPOx homopolymer and partially reversible for PIPOx-*b*-PMeOx copolymer. As revealed by NMR, the phase transition in PEtOx-based copolymers solutions exists despite the absence of solution turbidity. It is very broad, virtually independent of the copolymer composition and reversible with some hysteresis. Two types of water molecules were detected in solutions of the diblock copolymers above the phase transition—“free” with long and “bound” with short spin–spin relaxation times *T*_2_. NOESY spectra revealed information about conformational changes observed already in the pre-transition region of PIPOx-*b*-PMeOx copolymer solution.

## 1. Introduction

The polymers that can change their properties upon external stimuli, such as changes in temperature, pH, ionic strength, magnetic field, and light irradiation, as well as by applying ultrasound or enzyme, reactive oxygen species (ROS), or glucose addition, are called “smart” or stimuli-responsive polymers [1,2,3,4,5,6]. Over the past decades, they have become one of the most intensively studied class of materials because of their potential biomedical applications (i.e., drug/gene/DNA delivery, biosensors, bio-imaging agents) or tissue engineering applications [1,7,8,9,10,11,12]. Among stimuli-responsive materials, the thermoresponsive polymers are extensively examined because they can be used in non-invasive treatment. In particular, many investigations focus on materials that exhibit phase separation in water with the lower critical solution temperature (LCST) [13,14]. In the systems based on this type of polymers, polymer chains are easily dissolved at room temperature and their chains occur in a random coil conformation (a soluble form). While heating above LCST, the chains collapse to form a compact globule (an insoluble form of the particles). Considering the biomedical applications, the polymers must be biocompatible, non-toxic, and exhibit phase separation in water with LCST ~293 K to 308 K [15]. As described in a recently published review by Jana and Uchman [16], poly(2-oxazoline)s (POx)s meet these conditions. In general, water solution properties of POx are controlled by a side chain of the polymer. Increasing the size (length) of a side chain, the following dependence could be observed: short poly(2-methyl-2-oxazoline) (PMeOx) is hydrophilic and well soluble in water, poly(2-ethyl-2-oxazoline) (PEtOx, LCST ≈ 338 K), poly(2-isopropyl-2-oxazoline) (PIPOx, LCST ≈ 310 K), poly(2-cyclopropyl-2-oxazoline) (PCPOx, LCST ≈ 303 K), and poly(2-n-propyl-2-oxazoline) (PNPOx, LCST ≈ 297 K) exhibit thermoresponsive LCST-type phase transition in aqueous solutions, where the respective LCST depends on the molecular weight of polymer chains, while polymers with longer alkyl or fluoroalkyl side chains are hydrophobic [17,18,19,20,21,22].

Usually, the studies of the solutions temperature behavior in LCST-type phase transition are conducted utilizing light scattering, cloud point measurement, differential scanning calorimetry, infrared (IR) spectroscopy, NMR spectroscopy, small angle X-ray scattering etc., [23,24,25,26,27,28]. Among these methods, NMR spectroscopy stands out, because it can provide quantitative information on the LCST phase separation behavior. By diffusion and relaxation time experiments, it is possible to follow changes in molecular motions of polymer and water in solution. Moreover, to clarify conformational problems of macromolecules the 2D ^1^H–^1^H NOESY spectra are extremely valuable [10,29,30,31,32,33].

In the present contribution, we applied ^1^H NMR spectroscopy, ^1^H spin–spin relaxation times (temperature and time dependences), and 2D nuclear Overhauser effect spectroscopy (NOESY) at various temperatures (applied only to copolymers) to study temperature-induced phase separation in aqueous solutions of PIPOx homopolymer and PMeOx-*b*-PEtOx (with different composition) and PMeOx-*b*-PIPOx copolymers. Because of the fact that PMeOx, PEtOx, and PIPOx are biocompatible and do not accumulate in the tissues, in principle these systems can be applied in biomedicine, drug and gene delivery systems [9,34,35,36,37].

## 2. Materials and Methods

### 2.1. Materials

2-methyl-2-oxazoline (MeOx), 2-ethyl-2-oxazoline (EtOx), methyl p-tosylate (MTS), calcium hydride (CaH_2_), and acetonitrile (AN) were received from Sigma-Aldrich (St. Louis, MO, USA). 2-isopropyl-2-oxazoline (IPOx) was received from Tokyo Chemical Industry co (Fakuya, Japan). Potassium hydroxide (KOH) and methanol were obtained from LachNer (Neratovice, Czech Republic). MeOx, EtOx, IPOx, and AN were distilled over CaH_2_. MTS was used as received.

### 2.2. Synthesis and Characterization

POX homopolymers and block copolymers were prepared by a sequential cationic ring-opening polymerization (ROP) in acetonitrile, initiated by MTS at 135 °C. The reaction was terminated by 1M solution of KOH in methanol. Briefly, e.g., in the synthesis of PMeOx_10_-*b*-PIPOx_90_ 0.17 g (1.96 mmol, 10 eq) MeOx, 10 mL of AN, and 37 mg MTS (0.1964 mmol, 1 eq) were added into Ace pressure tube, degassed with argon and heated in an oil bath at 135 °C for 75 min. The reaction mixture was cooled to room temperature and under argon 2.0 g (17.7 mmol, 90 eq) of IPOx was added. Subsequently, the mixture was polymerized for 2 h at 135 °C and terminated with a four-fold excess of KOH/MeOH. The final polymer was purified by precipitation into ether and by dialysis against water (MWCO 1kDa). The yield was 1.1 g. The product was isolated with lyophilization. In the case of homopolymer synthesis, 100 eq of monomer was used at the polymerization and no additional monomer was added. The polymerization reaction was proceeded at the same conditions (135 °C, 75 min), terminated by KOH/MetOH and the synthesized polymer was isolated and purified by the same route as in the case of copolymers.

^1^H NMR spectroscopy was used for calculation of values of the molar ratio of MeOx, EtOx, and IPOx units in copolymers (from integrated intensities of NMR signals of corresponding methyl protons). Size-exclusion chromatography (SEC) was applied for molecular weights (*M_w_* and *M_n_*) and molecular-weight dispersity determination for all prepared polymers (in a mixture of chloroform: isopropyl alcohol: triethylamine in volume ratio 94:4:2, 1.0 mL/min, detection by RI detector, calibration on poly(methyl methacrylate) standards). All these values are shown in Table 1.

### 2.3. NMR Measurements

Bruker Avance III 600 spectrometer operating at 600.2  MHz was utilized for temperature dependences of ^1^H NMR spectra. The measurements were performed with following parameters: 90° pulse width = 10 μs, relaxation delay 10 s, 16 scans, acquisition time 2.18 s. Before each measurement sample was kept for 15 min at the desired temperature. 2D ^1^H–^1^H NOESY NMR spectra, with total of 16 scans accumulated over 512 t_1_ (evolution time) increments with a relaxation delay of 10 s, were obtained with 4098 Hz spectral window in both frequency axes. Two different mixing times 200 ms and 600 ms were used. ^1^H spin–spin relaxation times *T*_2_ of HDO (temperature and time dependences) were recorded using the CPMG pulse sequence 90°_x_-(t_d_-180°_y_-t_d_)_n_-acquisition [38]. Two scans with the relaxation delay 100 s and acquisition time 2.84 s were applied. In all measurements, BVT 3000 temperature unit was utilized to maintain constant temperature within ±0.2 K in the range 295–360 K. D_2_O (Sigma, 99.9% of deuterium) polymers solutions (concentrations *c* = 0.5, 5 and 20 wt%) were filled into 5-mm NMR tubes, subsequently degassed and sealed under nitrogen.

### 2.4. Dynamic Light Scattering (DLS) Measurements

Chosen samples were measured using ALV-6010 correlator equipped with an ALV/CGS-8F goniometer, a 22 mW He-Ne laser with wavelength λ = 632.8 nm, and pair of avalanche photodiodes working in a pseudo-cross-correlation mode. 90° angle was used in all measurements. The obtained intensity correlation function *g*^2^(*t*) was analyzed using the REPES algorithm [39] with the inverse Laplace transformation:

$$g^{2}(t)=1+\beta {\left[\int A(t)\exp(-t/\tau )d\tau \right]}^{2}=1+\beta {\left[\sum_{i=1}^{n}A_{i}\exp(-t/\tau_{i})\right]}^{2}$$

where: *β*—an instrumental parameter, *t*—the delay time of the correlation function, *A*(*τ*)—yielding distribution of relaxation times *τ*. The relaxation time *τ* is related to the relaxation (decay) rate Γ and diffusion coefficient *D* by the equation:

$$\Gamma =\frac{1}{\tau }=Dq^{2}$$

where: *q*—the scattering vector (*q* = (4π*n*/λ)sin(θ/2) where θ is the scattering angle and *n* is the refractive index of the solvent).

The studied solutions were prepared in D_2_O (*c* = 5 wt%, i.e., the same as in most of NMR measurements) and stabilized overnight at ambient temperature. After that, the solutions were transferred to glass tubes and flame-sealed to prevent evaporation of the solvent.

## 3. Results and Discussion

### 3.1. DLS

**Figure 1:**
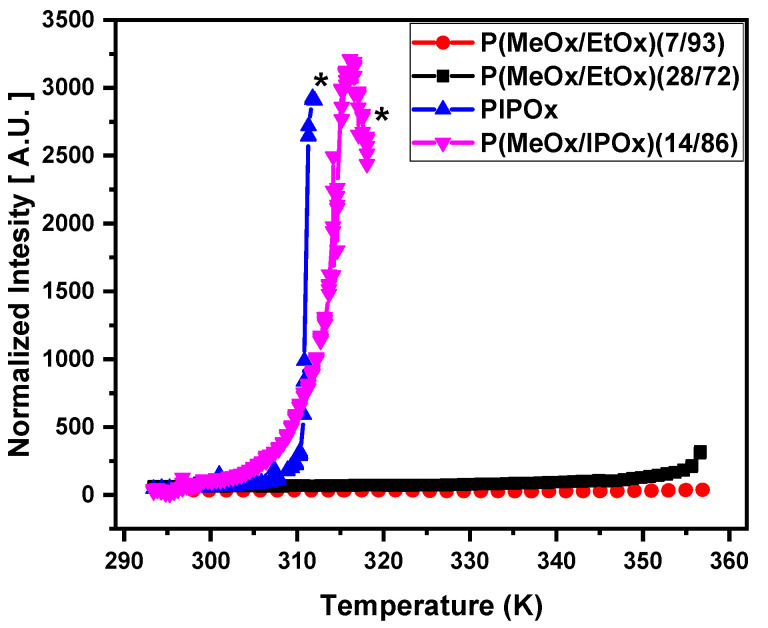
Temperature dependences of the intensity of scattered light in D_2_O solutions (*c* = 5 wt%) of PIPOx homopolymer, P(MeOx-*b*-EtOx) and P(MeOx-*b*-IPOx) copolymers during gradual heating. Symbol “*” is related to precipitation of the sample.

DLS was used for the study of the temperature behavior of D_2_O solutions (c = 5 wt%) of prepared PMeOx-*b*-PEtOx, PMeOx-*b*-PIPOx block copolymers, and PIPOx homopolymer on the macromolecular level. Temperature dependence of the normalized intensity of scattered light of all polymers is shown in Figure 1. The detected increase of the intensity is directly caused by an increase in the turbidity of the respective solutions. The significant difference is observed between PEtOx- and PIPOx-based samples. For PEtOx solutions, the turbidity was almost not detected by DLS, as well as by the visual observations. A similar effect was previously observed by our group for PEtOx-grad-PMeOx copolymers aqueous solutions [40]. On the other hand, in PIPOx homopolymer and PMeOx-*b*-PIPOx block copolymer solutions, a rapid increase of intensity of the scattered light is detected with LCST = 311 K and 313 K, respectively. A slightly higher LCST value for copolymer was expected due to the well-known effect that the addition of hydrophilic monomer units to thermoresponsive ones causes an increase in LCST of resulting copolymer [19,41]. These results are consistent with cloud point measurements obtained recently in the literature [20,42].

### 3.2. ^1^H NMR Spectra and Fraction p of Proton Groups (units) with Significantly Reduced Mobility

**Figure 2:**
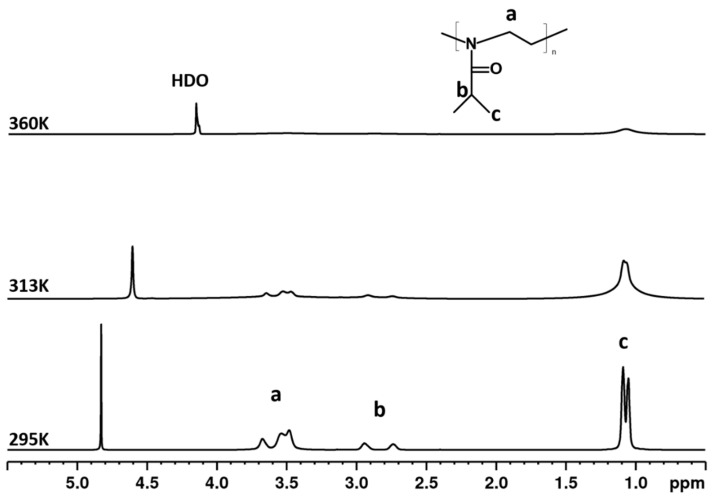
600.2 MHz ^1^H NMR spectra of PIPOx homopolymer in D_2_O solution (*c* = 5 wt%) measured at 295, 313, and 360 K under the same instrumental conditions.

Figure 2 shows high-resolution ^1^H NMR spectra of a D_2_O solution (*c* = 5 wt%) of the PIPOx homopolymer measured under the same instrumental conditions at three temperatures: below the LCST (295 K), in the middle of the transition (313 K) and above the LCST (360 K). The assignment of resonances to various proton types is shown directly in the spectrum measured at 295 K and the chemical structure of homopolymer is shown in the figure. The “a” signal is related to methylene protons NCH_2_ from the main chain of PIPOx, while PIPOx side chain groups COCH and CH_3_ correspond to peaks “b” and “c,” respectively. At first glance, when comparing the spectra at different temperatures, a broadening and disappearance of polymer signals with increasing temperature are observed. This result is definitely caused by the fact that with increasing temperature the mobility of polymer chains which create globular-like structures decreases to such an extent that they are not anymore detected in high-resolution NMR spectra. Similar behavior was previously observed for other thermoresponsive polymer-based systems e.g., poly(N-isopropyl acrylamide) (PNIPAm) [43], poly(N-vinylcaprolactam) (PVCL) [44], poly [2-(2-methoxyethoxy)ethyl methacrylate] (PDEGMA) [30], and PIPOx [29].

**Figure 3:**
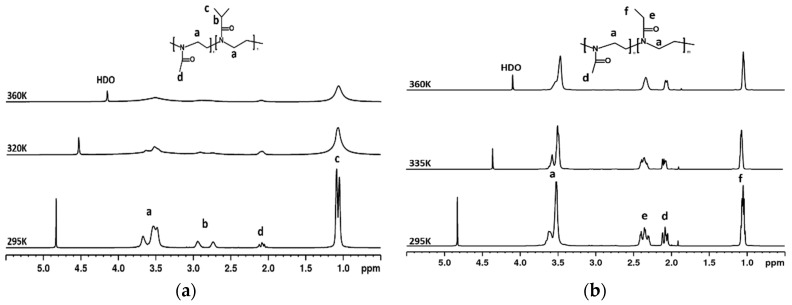
600.2 MHz ^1^H NMR spectra of P(MeOx/IPOx)(14/86) (**a**) and P(MeOx/EtOx)(28/72) (**b**) copolymers in D_2_O solutions (c = 5 wt%) measured at 295, 320, or 335 and 360 K under the same instrumental conditions.

High-resolution ^1^H NMR spectra of D_2_O solutions (c = 5 wt%) of P(MeOx/IPOx)(14/86) copolymer recorded under the same instrumental conditions at three temperatures 295 K, 320 K, and 360 K are shown in Figure 3a. Similarly as in Figure 2, the structure of copolymer and signal assignments of the various proton types are shown in the spectrum. Signals of IPOx units (“a, b, c”) are at the same positions as in spectra of PIPOx homopolymer (Figure 2). Additionally, a signal of methyl protons from MeOx units (“d”) is detected. On the spectra measured at 320 and 360 K, a similar effect to the homopolymer is observed. All copolymer signals become broader and almost disappear with the increasing temperature. As the signal “d” of hydrophilic MeOx units vanishes at high temperatures, it excludes the core-to-shell formation above LCST, which was observed previously for PNIPAm-*b*-PEO systems [45]. In Figure 3b high-resolution ^1^H NMR spectra of D_2_O solutions (c = 5 wt%) of P(MeOx/EtOx) (28/72) copolymer recorded under the same instrumental conditions at three temperatures 295 K, 335 K, and 360 K are shown. Likewise as for two previous cases, the structure of copolymer and signal assignments of the various proton types are shown in the spectrum. The signal “a” is related to methylene protons NCH_2_ from the copolymer backbone, while PEtOx side chain groups COCH_2_ and CH_3_ correspond to peaks “e” and “f,” respectively. Moreover, the signal of methyl protons from MeOx units (“d”) is detected at the same position as in the spectrum of P(MeOx/IPOx)(14/86) copolymer. As it was abovementioned for PIPOx-based samples, a decrease in integral intensity of all copolymer signals is observed, but contrary to them this effect is much weaker. A similar result was detected for P(MeOx/EtOx)(7/93) copolymer. This is in accordance with our previous observations obtained for PEtOx-grad-PMeOx copolymers aqueous solutions [40].

**Figure 4:**
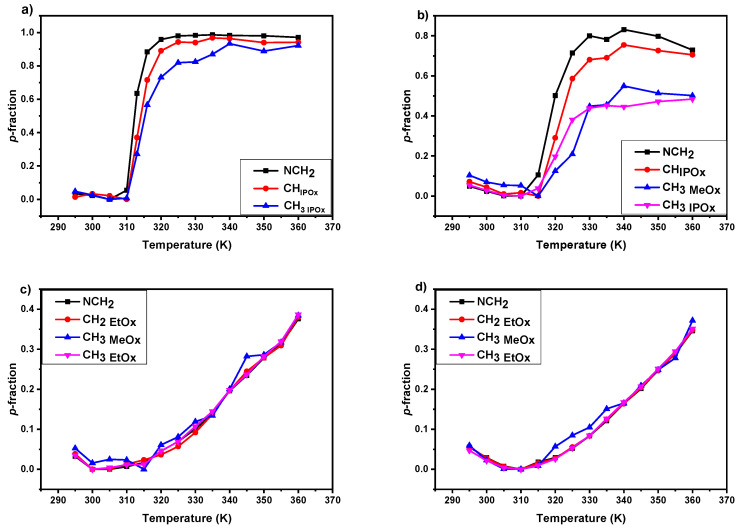
Temperature dependences of the fraction *p* as determined for all signals of various proton types in D_2_O solutions (*c* = 5 wt%) of PIPOx homopolymer (**a**) and P(MeOx/IPOx)(14/86) (**b**), P(MeOx/EtOx)(28/72) (**c**), P(MeOx/EtOx)(7/93) (**d**) copolymers during gradual heating.

For the quantitative characterization of changes that occur during the heating and cooling processes, temperature-dependent integrated intensities of NMR signals were used. For this purpose, the values of the fraction *p* of proton groups of the given type with significantly reduced mobility were obtained using the relation [27,38,40]:

(1) $$p=1-\frac{I(T)}{I(T_{0})\times \frac{T_{0}}{T}}$$

where: *I(T)*—the integrated intensity of respective polymer signal at given absolute temperature *T*, *I(T_0_)*—the integrated intensity of this signal in the case of no phase transition (or other reason for the polymer segments mobility reduction). The temperature where the integrated intensity of the given signal was the highest was set as *T_0_* (therefore *p*(*T*_0_) = 0). Moreover, the fact that the integrated intensities should decrease with temperature as 1/*T* was taken into account in the denominator of the Equation (1). In Figure 4, temperature dependences of the *p*-fraction of various proton types of all D_2_O solutions (*c* = 5 wt%) are shown. There is a visible difference in temperature behavior between IPOx- and EtOx-based samples. In the case of PIPOx homopolymer and P(MeOx/IPOx)(14/86) copolymer, for all proton groups *p*-fraction first slightly decreases and has a minimum at ≈ 310 K. At temperatures above 310 K the values of the *p*-fraction are increasing drastically, and the phase transition occurs in accord with DLS results (see Figure 1). Temperature dependences of the *p*-fraction determined from integrated intensities of various PIPOx signals are slightly different: *p*-fraction values of polymer backbone (NCH_2_) increases faster than polymer side-chain (CH_IPOx_, CH_3 IPOx_). This means that polymer main-chains first interact with each other, after that side-chain follows them similarly restricted in mobility and PIPOx homopolymer forms aggregates. The maximum values of the *p*-fraction (*p*_max_ ≈ 1) give quantitative information on the fraction of polymer chains which participates in the phase transition and have been achieved around 340 K. The LCST (defined as the temperature at *p*_max_/2) was estimated for PIPOx as 312 K, which is in agreement with DLS (311K) results when we consider the temperature in the middle of the transition interval (Figure 1). In temperature dependences of the *p*-fraction determined for P(MeOx/IPOx)(14/86) copolymer this effect is more visible, values of *p*-fraction of copolymer backbone increase first and show a sharp transition with a high *p*_max_ (≈0.80) and LCST shifted to ≈318 K. But from the side-chains, only the CH_IPOx_ shows a sharp transition with corresponding transition parameters (*p*_max_ ≈ 0.75, LCST ≈ 320K). The rest of the side chain proton groups (hydrophilic CH_3 MeOx_, thermoresponsive CH_3 IPOx_) reach a smaller *p*_max_ (≈0.40) value at 330 K with higher LCST ≈ 323 K and 325 K for CH_3 IPOx_ and CH_3 MeOx,_ respectively. This effect can suggest the formation of a fiber-like structure with an immobilized core formed from copolymer main-chains and partially mobile (60% of MeOx and IPOx methyl groups are still mobile at temperatures above phase transition) side-chain corona observed previously by Legros et al. [46]. The described behavior was previously observed for PDEGMA-*b*-PHPMA copolymers [30], but also it is in contrast to various thermoresponsive homopolymers and copolymers based on PNIPAm, PVCL, or PEtOx [38,40,45,47,48]. In contrast to IPOx, in EtOx-based copolymers, from the temperature dependences of the fraction *p* determined from integrated intensities of all copolymer signals, virtually the same way of transition is observed for the main chain and side chains of both hydrophilic MeOx and thermoresponsive EtOx units. Additionally, in both cases they reach the smaller *p*_max_ (≈0.40) values at 360 K. Practically the same behavior was obtained for P(MeOx-*grad*-EtOx) gradient copolymers aqueous solutions [40]. In further considerations, we will use *p*-fraction temperature dependences of NCH_2_ protons, because of its presence in all prepared polymers.

**Figure 5:**
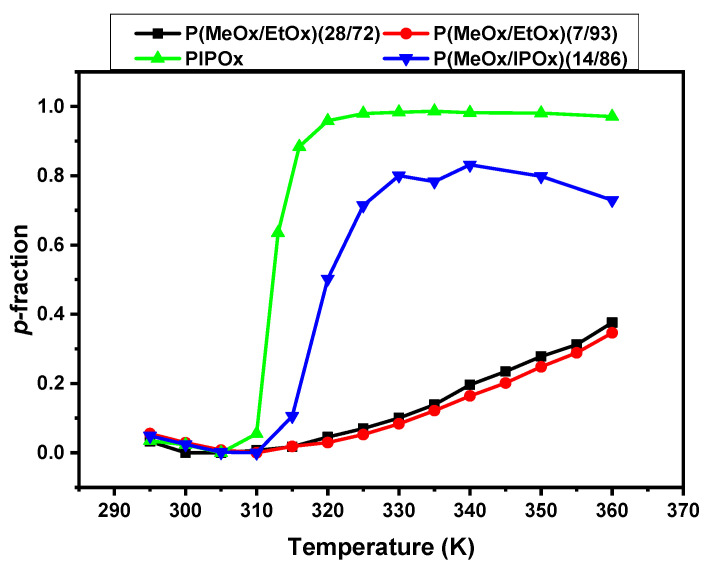
Temperature dependencies of the fraction *p* of units with significantly reduced mobility for NCH_2_ protons in D_2_O solutions (*c* = 5 wt%) of PIPOx homopolymer (green) and P(MeOx/IPOx)(14/86) (blue), P(MeOx/EtOx)(28/72) (black), P(MeOx/EtOx)(7/93) (red) copolymers during gradual heating.

Temperature dependences of the fraction *p* of D_2_O solutions (*c* = 5 wt %) of all investigated polymers are shown in Figure 5. As it was abovementioned, PIPOx homopolymer has a quite sharp (transition width 8 K) and complete (*p*_max_ ≈ 1) phase transition with LCST ≈ 312 K. The addition of hydrophilic MeOx units in the form of a block, results in a broader (transition width increases to ≈15 K), incomplete (*p*_max_ ≈ 0.80) phase transition with higher LCST ≈ 318 K. All of these effects, caused by the addition of hydrophilic block to the thermoresponsive one, were observed in PNIPAm-*b*-PEO and PDEGMA-*b*-PHPMA water solutions [30,45]. Additionally, the increase in the LCST value upon the addition of hydrophilic components is in agreement with DLS results (see Figure 1), as well as with results presented in the literature for different propyl-oxazolines systems [19,20,41]. In contrast to PIPOx-based polymers, the phase transition in D_2_O solutions of P(MeOx-*b*-EtOx) copolymers is very broad, *p*-values gradually increase from 310 K without any noticeable jump (transition width at least ≈ 50 K). In addition, values of *p*_max_ are rather low (*p*_max_ = 0.40). Moreover, the temperature dependences of the *p*-fraction are almost independent of copolymer composition. Also interesting is the fact that no significant changes (no turbidity) are observed by DLS in the temperature dependence of the normalized intensity of scattered light. A very similar effect of a very broad transition and low *p*_max_-values was observed for PEtOx-based block and gradient copolymers [38,40].

**Figure 6:**
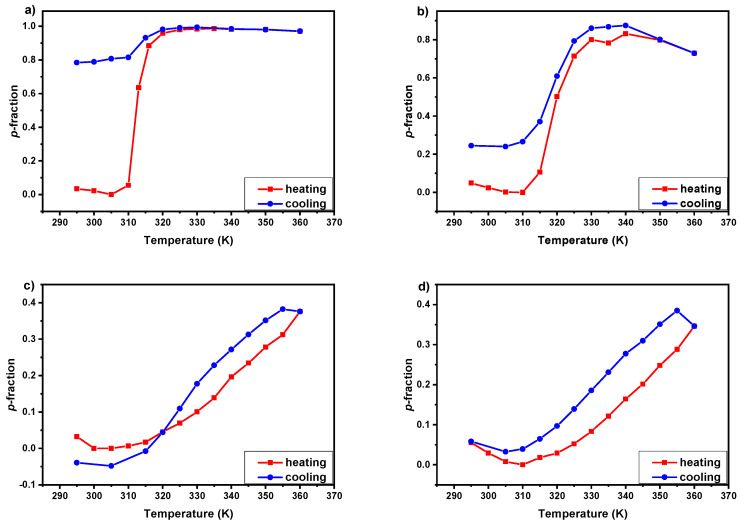
Temperature dependences of the fraction *p* of units with significantly reduced mobility for NCH_2_ protons in D_2_O solutions (*c* = 5 wt%) of PIPOx homopolymer (**a**) and P(MeOx/IPOx)(14/86) (**b**), P(MeOx/EtOx)(28/72) (**c**), P(MeOx/EtOx)(7/93) (**d**) copolymers during gradual heating and subsequent gradual cooling.

To study the reversibility of the phase transition, directly after the heating process analogous measurements during subsequent gradual cooling were provided. In Figure 6 temperature dependences of the fraction *p* of units with significantly reduced mobility for NCH_2_ protons in D_2_O solutions (*c* = 5 wt%) of PIPOx homopolymer (a) and P(MeOx/IPOx)(14/86) (b), P(MeOx/EtOx)(28/72) (c), P(MeOx/EtOx)(7/93) (d) copolymers during gradual heating and subsequent gradual cooling are shown. Generally, similarly to the heating, the behavior of all proton groups of the main-chain and the side-chains is the same in almost all cases (Figures S1, S3 and S4 in Supplementary Materials), only the methyl group of MeOx units in P(MeOx/IPOx)(14/86) copolymer is almost fully reversible, in contrast to the rest of copolymer proton groups (Figure S2 in Supplementary Materials). As is observed in Figure 6a, in the case of PIPOx homopolymer the *p*-fraction values just slightly decrease (from 1 to 0.80) during gradual cooling to temperatures below LCST. This means that in 80% the structures formed during the heating are preserved during cooling, and display irreversibility of the phase transition. Moreover, the sample was turbid after lifting out from the magnet, which is in agreement with this result. The irreversibility of the phase transition (even temperature induce crystallization) of PIPOx-based aqueous solutions was observed previously in the literature [49,50,51,52]. Different behavior was observed for P(MeOx/IPOx)(14/86) copolymer solution (Figure 6b). Herein, during gradual cooling, values of the *p*-fraction decrease with slight hysteresis (from 0.80 to 0.30 for) in temperatures below LCST, more than in PIPOx homopolymer. Same as for PIPOx, the sample was also turbid after lifting out from the magnet. In this case, we can assume that the phase transition of P(MeOx/IPOx)(14/86) copolymer is partially reversible. A similar effect was observed by Legros et al. for P(MeOx_50_-*b*-IPOx_50_) copolymer water solutions studied by DLS [46]. The authors observed reversible self-assembly only for solutions kept for a short time (less than 90 min), but for longer incubation above LCST the process becomes irreversible. In our case, considering the number of temperature points measured above LCST (7), time of the single ^1^H NMR experiment (3 min), as well as the time consumed for temperature change and stabilization (≈20 min), the solution was kept in temperature above LCST around 3 h. This time was probably enough for the partial crystallization of polymer chains (≈60%). In contrast to PIPOx-based polymers, in both P(MeOx/EtOx) copolymer solutions changes in the *p*-fraction values are completely reversible, as shown in Figure 6c,d. In both cases, gradual cooling measured directly after previously heating results in the decrease of the *p*-fraction values with well-visible hysteresis, which was previously reported for P(MeOx-*grad*-EtOx) gradient copolymers aqueous solutions [40].

**Figure 7:**
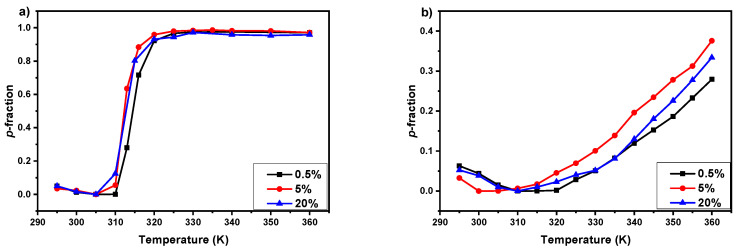
Temperature dependences of the fraction *p* of PIPOx homopolymer (**a**) and P(MeOx/EtOx)(28/72) copolymer (**b**) in D_2_O solutions with three polymer concentrations (*c* = 0.5; 5; 20 wt%) during gradual heating.

Based on the earlier investigations of IPOx- and EtOx-based polymer systems, which show that LCST depends on polymer concentration [19,20,23,40], we decide to study PIPOx homopolymer and P(MeOx/EtOx)(28/72) copolymer solutions with three concentrations (*c* = 0.5, 5, and 20 wt%). In Figure 7, temperature dependences of the fraction *p* of PIPOx homopolymer (a) and P(MeOx/EtOx)(28/72) copolymer (b) in D_2_O solutions with three polymer concentrations (*c* = 0.5; 5; 20 wt%) during gradual heating are shown. Similarly to samples with 5 wt% concentration (Figure 4), also in 0.5 and 20 wt% polymer solutions, all polymer proton types show virtually the same temperature dependences of the *p*-fraction (Figures S5–S8). In the case of PIPOx homopolymer solutions (Figure 7a), the small dependence of LCST on concentration is observed. This difference is visible especially for the samples with 0.5 and 5 wt% concentration, which for lower concentration exhibit higher LCST (314 K) than for 5 wt% solution (LCST = 312 K). Interesting is the fact that the increase in PIPOx concentration to 20 wt% does not lead to a further decrease in LCST values. The curves of phase transition for 5 and 20 wt% PIPOx solutions are practically identical. The same effect of concentration for PIPOx homopolymer aqueous solutions was observed using cloud point measurements by Hijazi et al. [20]. They show that LCST of PIPOx is decreasing with temperature up to 5 wt% and remains stable up to 50 wt% concentration. Temperature dependences of the fraction *p* of P(MeOx/EtOx)(28/72) copolymers solutions also show the effect of concentration on LCST values. Herein, the solution with 5 wt% concentration exhibits the lowest LCST value (≈340 K) in comparison to 0.5 and 20 wt% (LCST≈ 345K). However, it is too small range of concentrations to definitively state, which type of phase transition it is [53]. This behavior is in contrast to P(MeOx-*grad*-EtOx) gradient copolymers aqueous solutions, where LCST values are decreasing with increasing concentration [40].

### 3.3. Spin–Spin Relaxation Times T_2_ of Water (HDO) Molecules

**Figure 8:**
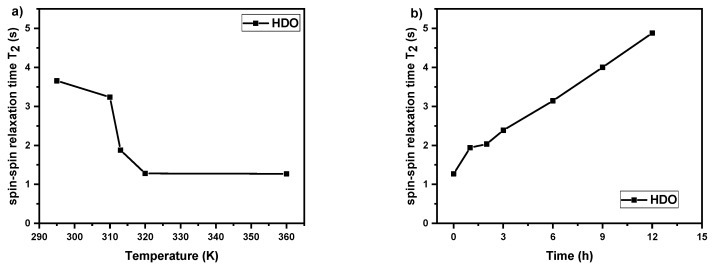
Temperature dependence (**a**) and time dependence at 360 K (**b**) of ^1^H spin–spin relaxation times *T*_2_ of HDO in D_2_O solution (*c* = 5 wt%) of the PIPOx homopolymer.

In order to characterize changes in polymer-solvent interactions, ^1^H spin–spin relaxation times T_2_ of water (HDO protons) measurements were conducted. As it was shown in the literature for various thermoresponsive homopolymers and copolymers in aqueous solutions, these studies can give valuable information on the behavior of water molecules during LSCT type phase transition [27,30,33,40,45]. We measured ^1^H spin–spin relaxation times T_2_ of water molecules for all polymers solutions with 5 wt% concentration with temperature and time dependences (at 360 K). Temperature dependence experiments were done at the temperature points based on the of the *p*-fraction values (Figure 5). At all temperatures, there was a single line of HDO in ^1^H NMR spectrum and this holds for all investigated samples. In Figure 8a temperature dependence of ^1^H spin–spin relaxation time *T*_2_ of HDO in D_2_O solution (*c* = 5 wt%) of the PIPOx homopolymer is presented. The starting value of *T*_2_ (≈3.6 s) is related to the mobility of water molecules in solution and implies that water molecules interact with PIPOx polymer chains by hydrogen bonding. Additionally, *T*_2_ values slightly decrease with temperature to the starting point of PIPOx phase transition, which suggests some changes in the interactions in solution already at this temperature. Subsequently, *T*_2_ values significantly decrease in the area of the phase transition to *T*_2_ = 1.28 s at 320 K (temperature directly above PIPOx phase transition). This shows that during the phase transition there is some portion of HDO molecules that are bound (confined) in a rather compact structure formed by PIPOx chains. This effect has been maintained also at 360 K. Next, at 360 K *T_2_* values start to increase with time (Figure 8b) reaching *T*_2_ = 4.88 s after 12 h. This indicates that during this time, water molecules are “released” from polymer aggregates. Probably, in aggregates, polymer–polymer interactions become stronger with time to such extent that polymer chains exhibit the irreversible phase transition and crystallization. The “releasing” of water molecules with time at a temperature above the LCST phase transition was observed for other thermoresponsive polymer systems based on PNIPAm or PVCL [45,48]. Moreover, monoexponential *T*_2_ relaxation curves even above the transition region demonstrate the “bound” water by reduced *T*_2_ values of HDO. Therefore, in this case there is a fast exchange between “bound” and “free” sites and the measured relaxation rates *T*_2_^−1^ are then given as a weighted average of the relaxation rates of bound and free HDO [40,45,54].

**Figure 9:**
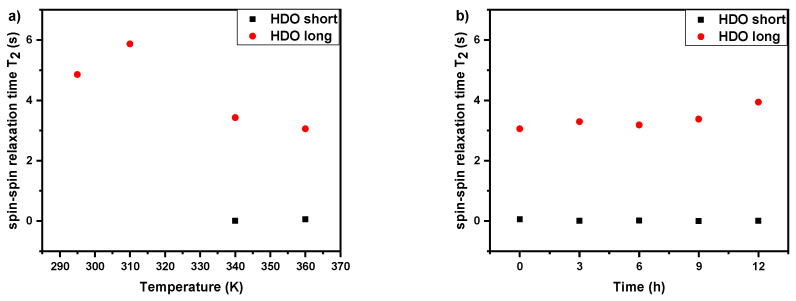
Temperature dependence (**a**) and time dependence at 360 K (**b**) of ^1^H spin–spin relaxation times *T*_2_ of HDO in D_2_O solution (*c* = 5 wt%) of the P(MeOx/EtOx)(7/93) copolymer.

In contrast to PIPOx homopolymer solution, in copolymer solutions different behavior of water molecules is observed. In Figure 9, Figures S9 and S10 temperature and time dependences of ^1^H spin–spin relaxation time *T*_2_ of HDO in D_2_O solutions (*c* = 5 wt%) of the copolymers P(MeOx/EtOx)(7/93), P(MeOx/IPOx)(14/86), and P(MeOx/EtOx)(28/72) respectively are shown. Generally, at temperatures in the middle (P(MeOx/EtOx)(7/93), P(MeOx/EtOx)(28/72)) and above phase transition (all three cases), the relaxation curves were bi-exponential and two *T*_2_ components were necessary to fit experimental relaxation curves well. In more details, for (P(MeOx/EtOx)(7/93) copolymer solution we observe the existence of two types of water at temperature *T* = 340 K. The first type is “free water” (HDO molecules in solution) with longer relaxation times (*T*_2_ ≈ 3 s) and the second type is “bound” water, with *T*_2_ values which are 3 orders of magnitude shorter (*T*_2_ ≈ 10 ms at 340 K), which represent HDO molecules inside the nanoparticles or aggregates formations. This effect remains also at 360 K. A similar phenomenon was previously observed for PDEGMA-*b*-PHPMA block copolymers, as well as for PEtOx homopolymers and PEtOx-based gradient or block copolymers [30,38,40].

### 3.4. 2D ^1^H-^1^H NOESY NMR Spectra

**Figure 10:**
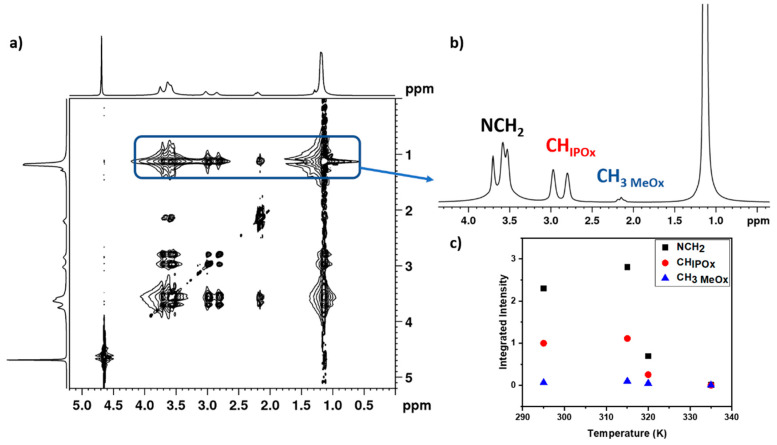
2D NOESY spectrum of P(MeOx/IPOx)(14/86) block copolymer in D2O solution (*c* = 5 wt%) measured at 315 K with mixing time 600 ms (**a**). On the right up (**b**) there is 1D slice spectrum extracted from the signal at 1.05 ppm of CH_3_ protons of PIPOx units of the NOESY spectrum. Temperature dependences of integrated intensities of various signals in 1D slices extracted from the signal of CH_3_ protons of PIPOx units (at 1.05 ppm) of the NOESY NMR spectra (**c**).

To obtain information on spatial proximity between proton groups of PMeOx and PIPOx (PEtOx) units, as well as to understand the conformational changes occurring during the phase separation, 2D nuclear Overhauser effect spectroscopy (NOESY) was employed [10,55,56]. We choose experimental parameters (especially the mixing time) of the NOESY NMR measurements based on the literature and our previous studies of other thermoresponsive polymer systems [10,30,40,45,56]. For this investigations two samples were chosen: P(MeOx/IPOx)(14/86) and P(MeOx/EtOx)(28/72) copolymers solutions (*c* = 5 wt%). In case of P(MeOx/IPOx)(14/86) copolymer, 2D ^1^H–^1^H NOESY NMR spectra were measured at four temperatures: at 295 K (starting temperature, below the transition, Figure S11), 315 K (temperature directly below the transition, Figure 10a), 320 K (in the middle of the transition, Figure S12), and 335 K (above the transition, Figure S13).

In NOESY spectra not only cross-peaks between various proton groups within PMeOx or PIPOx units were detected, but also weaker cross-peak between side chain CH_3_ protons of PIPOx units (signal at 1.05 ppm) and PMeOx side chain protons (at 2.1 ppm Figure 10). The presence of these cross-peaks implies that the distances between respective protons are smaller than 0.5 nm. Both PIPOx and PMeOx units that are in close proximity can be from the same chain of the copolymer, assuming a random-coil conformation of copolymer chains, but also from different copolymer chains. To quantitatively characterize the changes occurring with temperature, we used integrated intensities of signals of PIPOx and MeOx proton groups in 1D slices extracted from the signal of CH_3_ protons of PIPOx units at 1.05 ppm of the NOESY spectra. These 1D slices are shown in the right part of Figure 10 and Figures S11–S13. In Figure 10c temperature dependences of the absolute integrated intensities in slices extracted from the signal of CH_3_ protons of PIPOx units measured with mixing time 600 ms for D_2_O solution (*c* = 5 wt%) of the P(MeOx/IPOx)(14/86) copolymer are presented. From this dependence it follows that dependences of the intensities of the main chain NCH_2_ as well as the side chain CH_IPOx_ and CH_3 MeOx_ protons show a maximum at 315 K and then decrease almost to zero. The decrease in the intensity of these protons in the transition region (temperatures >315 K, see Figure 4b) is evidently in connection with the significantly reduced mobility of copolymer segments that form compact aggregates. The increased intensity of the respective copolymer signals at 315 K in comparison with 295 K shows that average distance between PIPOx protons and respective PMeOx protons is smaller at 315 K than at 295 K, and/or that number of close contacts between PIPOx and respective PMeOx proton groups is increased at 315 K. This indicates a change in conformation of the block copolymer. What is important is the fact that this change occurs directly at the temperature corresponding to the starting point of the LCST transition of the copolymer. Therefore, Figure 10c reveals information about certain conformation changes in the P(MeOx/IPOx)(14/86) block copolymer already in the pre-transition region. A similar effect was observed for PEO-*b*-PNIPAm block copolymer aqueous solution [45]. On the other hand, for P(MeOx/EtOx)(28/72) copolymer solution (c = 5 wt%) we do not detect any cross-peak between CH_3_ side chain protons of PEtOx units (signal at 1.05 ppm) and PMeOx CH_3_ side chain protons (Figures S14–S16). This suggests that there are no PEtOx and PMeOx units, which are in close proximity in the solution. This result is in contrast to P(MeOx-*grad*-EtOx) gradient copolymers aqueous solutions, where probably because of the structure of copolymer chain such signal was observed [40].

## 4. Conclusions

In summary, herein we report temperature behavior of thermoresponsive PIPOx homopolymer as well as PIPOx- and PEtOx-based diblock copolymers in D_2_O solutions investigated by ^1^H NMR methods, which were compared with DLS measurements. A combination of ^1^H NMR, ^1^H spin–spin relaxation time *T*_2_ and 2D ^1^H–^1^H NOESY measurements were used for the characterization of the structural changes on the molecular level and behavior of water and copolymer molecules during the temperature-induced phase transition.

We studied one PIPOx homopolymer, one P(MeOx/IPOx)(14/86) copolymer, and P(MeOx/EtOx)(28/72), P(MeOx/EtOx)(7/93) copolymers aqueous solutions. By DLS sharp phase transition for PIPOx-based samples dependent on polymer composition were observed, while almost no turbidity was detected for PEtOx-based copolymers.

Temperature dependences of fraction *p* of units with significantly reduced mobility, as determined from NMR spectra also show a sharp composition dependent phase transition for PIPOx-based polymers. In the case of PIPOx homopolymer solutions, phase transition was irreversible and depends on polymer concentration, while for P(MeOx/IPOx)(14/86) copolymer solution phase transition was partially reversible with small hysteresis. On the other hand, for PEtOx-based copolymers solutions, the phase transition is very broad and weak (low *p*_max_-values) so that even the term “transition” seems to be unsuitable. Additionally, it is almost independent of the copolymer composition and only slightly dependent on the concentration of the solution.

^1^H spin–spin relaxation times *T*_2_ of HDO experiments show different behavior of the water molecules in homopolymer and copolymer solutions. While in case of PIPOx homopolymer solution the single *T*_2_ values decrease with temperature and increase with time at the temperature above LCST (360 K), in the case of copolymers solutions two types of water, “free” and “bound” with long and very short *T*_2_ values, respectively, were detected at temperatures in the transition region and above the transition. At 360 K these two types of water stay unchanged for at least 12 h without release of the “bound” water. The detected two *T*_2_ components demonstrate that the exchange between “free” and “bound” water molecules is slow considering *T*_2_ values (residence time of the “bound” HDO ≥ 0.1 s).

Information on spatial correlations between protons of PIPOx and MeOx units was obtained from temperature dependences of 2D NOESY spectra. We detected not only cross-signals between NCH_2_ main chain and CH_3_ MeOx side chain protons, but also CH and CH_3_ PIPOx units. After changing the temperature from 295 to 315 K, increasing of the integrated intensity of signals from both units was observed in 1D slices extracted from 2D NOESY spectra. These results demonstrate that the average distance between PMeOx and PIPOx protons decreases at 315 K, and/or there is significant increase of the number of close contacts (<0.5 nm) between blocks at 315 K. The both cases evidence the change in the block copolymer conformation. As this change appears at the temperature, which is directly below the LCST transition of copolymer, certain conformation changes occur already in the pre-transition region.

This study emphasizes the importance of understanding the self-association of polymers in solution on the molecular level and provides important information for the design of “smart” thermoresponsive polymer-based drug delivery systems.

## Figures and Tables

**Table 1:** Molecular characteristics of the IPOx homopolymer and block copolymers.

Sample	*M_n_* [g/mol]	*M_w_* [g/mol]	*Ð*	MeOx/EtOx(IPOx) NMR *	MeOx/EtOx(IPOx) (eq) **
P(MeOx/EtOx)(28/72)	10,200	11,800	1.16	28/72	25/75
PIPOx	13,000	13,900	1.07	0/100	0/100
P(MeOx/IPOx)(14/86)	13,000	13,700	1.05	14/86	10/90
P(MeOx/EtOx)(7/93)	7300	8100	1.10	7/93	10/90

* Molar ratio in the copolymer determined by ^1^H NMR spectra. ** Monomer ratio in the synthesis.

## Supplementary Materials

The following are available online at https://www.mdpi.com/2073-4360/12/9/1879/s1. Figures S1–S8: Temperature dependences of the *p-* fraction. Figures S9 and S10: Temperature dependences and time dependences at 360 K of ^1^H spin–spin relaxation times *T*_2_ of HDO in D_2_O solutions. Figures S11–S15: 2D NOESY spectra.
