# Supplementary material for: Temperature Behavior of Aqueous Solutions of Poly(2-Oxazoline) Homopolymer and Block Copolymers Investigated by NMR Spectroscopy and Dynamic Light Scattering
Source: Polymers (Basel). 2020 Aug 20;12(9):1879. doi: 10.3390/polym12091879 (PMC7565327; doi:10.3390/polym12091879)
Supplement: Supplementary file 1 [file polymers-12-01879-s001.pdf]

Supplementary material

Temperature behavior of aqueous solutions of poly(2-oxazoline) homopolymer and block copolymers investigated by NMR spectroscopy and dynamic light scattering

Rafał Konefał [1*], Peter Černoch [1], Magdalena Konefał[1] and Jiří Spěváček [1,&,*]

[1] Institute of Macromolecular Chemistry CAS, Heyrovského nám. 2, 162 06 Prague 6, Czech Republic; konefal@imc.cas.cz (R.K.), cernoch@imc.cas.cz (P.Č.),magdalenakonefal@imc.cas.cz (M.K.), spevacek@imc.cas.cz (J.S.),

* Correspondence: konefal@imc.cas.cz (R.K.), spevacek@imc.cas.cz (J.S.)

& Professor Emeritus (J.S.)

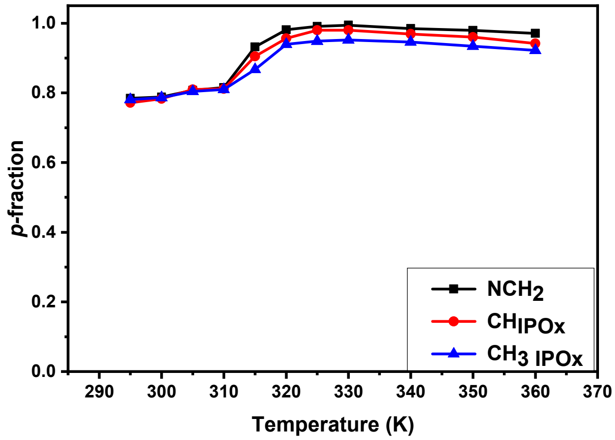

Figure S1. Temperature dependences of the fraction p as determined for all signals of various proton types in D_2O solution (c = 5 wt%) of PIPOx homopolymer during gradual cooling.

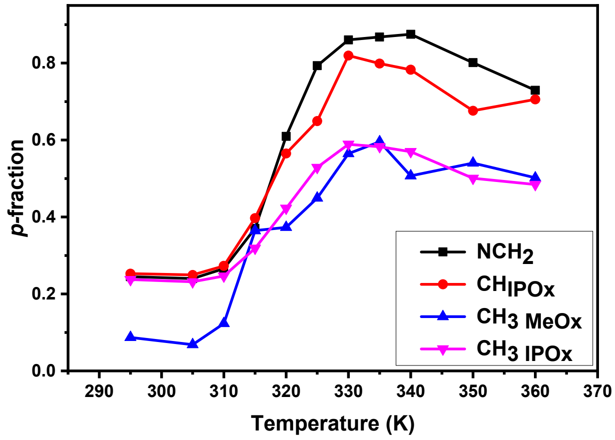

Figure S2. Temperature dependences of the fraction *p* as determined for all signals of various proton types in D_2O solution (*c* = 5 wt%) of P(MeOx/IPOx)(14/86) copolymer during gradual cooling.

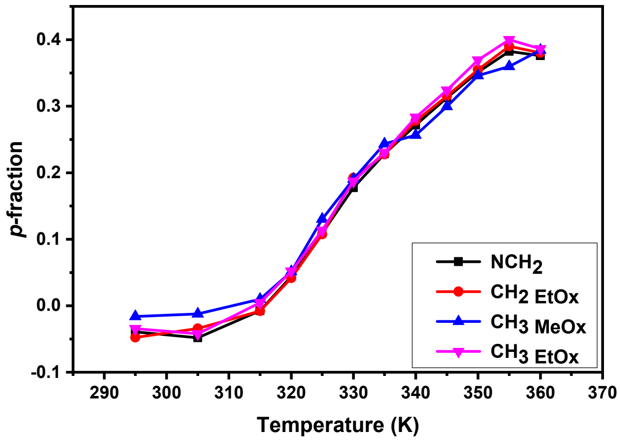

Figure S3. Temperature dependences of the fraction *p* as determined for all signals of various proton types in D_2O solution (*c* = 5 wt%) of P(MeOx/EtOx)(28/72) copolymer during gradual cooling.

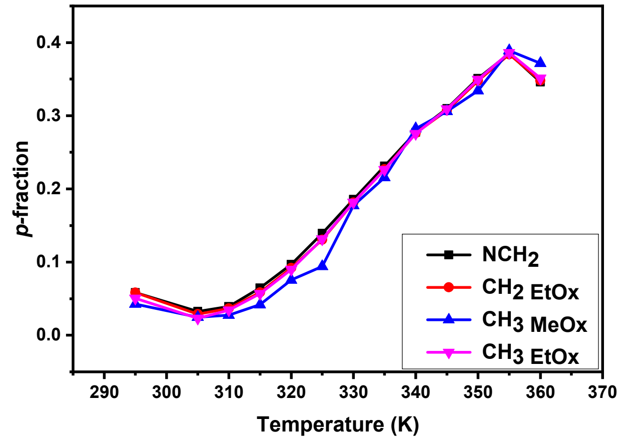

Figure S4. Temperature dependences of the fraction *p* as determined for all signals of various proton types in D_2O solution (*c* = 5 wt%) of P(MeOx/EtOx)(7/93) copolymer during gradual cooling.

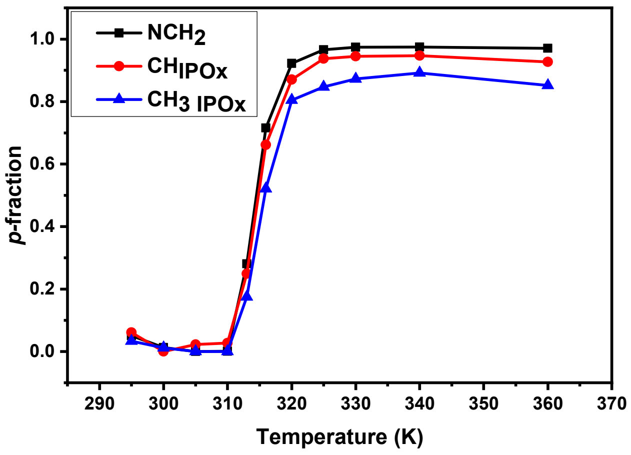

Figure S5. Temperature dependences of the fraction *p* as determined for all signals of various proton types in D_2O solution (*c* = 0.5 wt%) of PIPOx homopolymer during gradual heating.

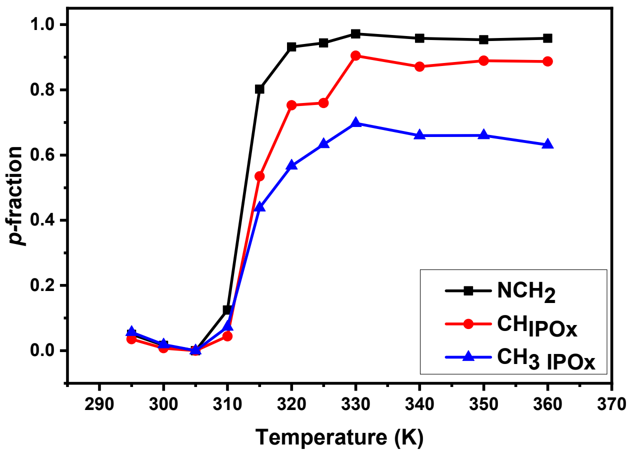

Figure S6. Temperature dependences of the fraction *p* as determined for all signals of various proton types in D_2O solution (*c* = 20 wt%) of PIPOx homopolymer during gradual heating.

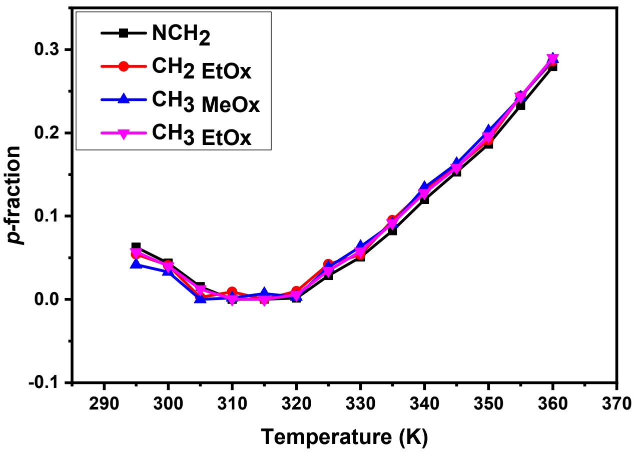

Figure S7. Temperature dependences of the fraction *p* as determined for all signals of various proton types in D_2O solution (*c* = 0.5 wt%) of P(MeOx/EtOx)(28/72) copolymer during gradual heating.

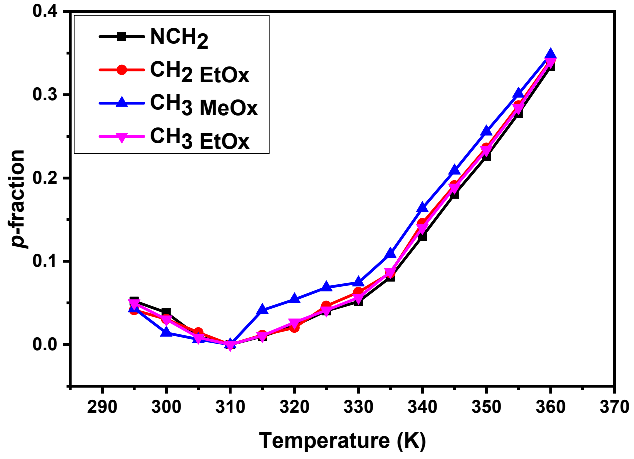

Figure S8. Temperature dependences of the fraction *p* as determined for all signals of various proton types in D_2O solution (*c* = 20 wt%) of the P(MeOx/EtOx)(28/72) copolymer during gradual heating.

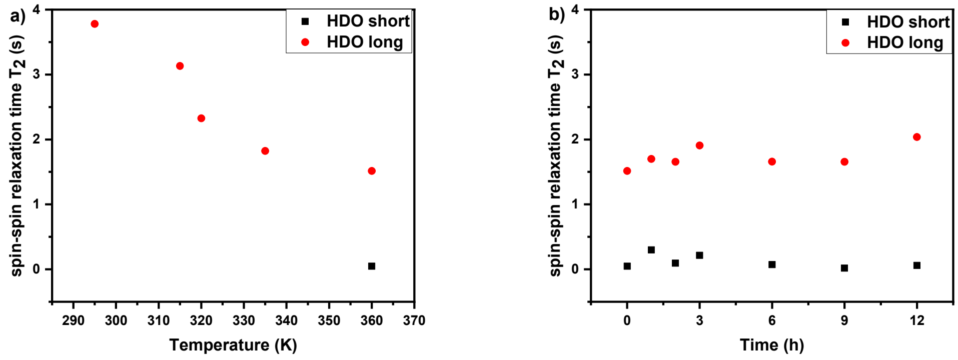

Figure S9. Temperature dependence (a) and time dependence at 360 K (b) of ^{1}H spin-spin relaxation times T_2 of HDO in D_2O solution (c = 5 wt%) of the P(MeOx/IPOx)(14/86) copolymer.

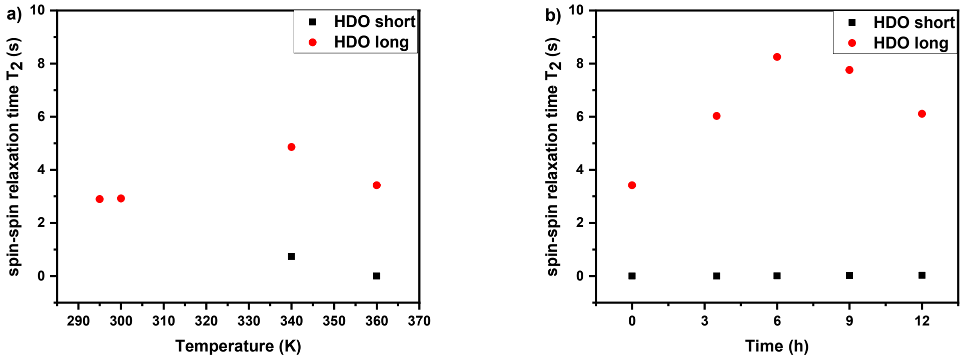

Figure S10. Temperature dependence (a) and time dependence at 360 K (b) of ^{1}H spin-spin relaxation times T_2 of HDO in D_2O solution (c = 5 wt%) of the P(MeOx/EtOx)(28/72) copolymer.

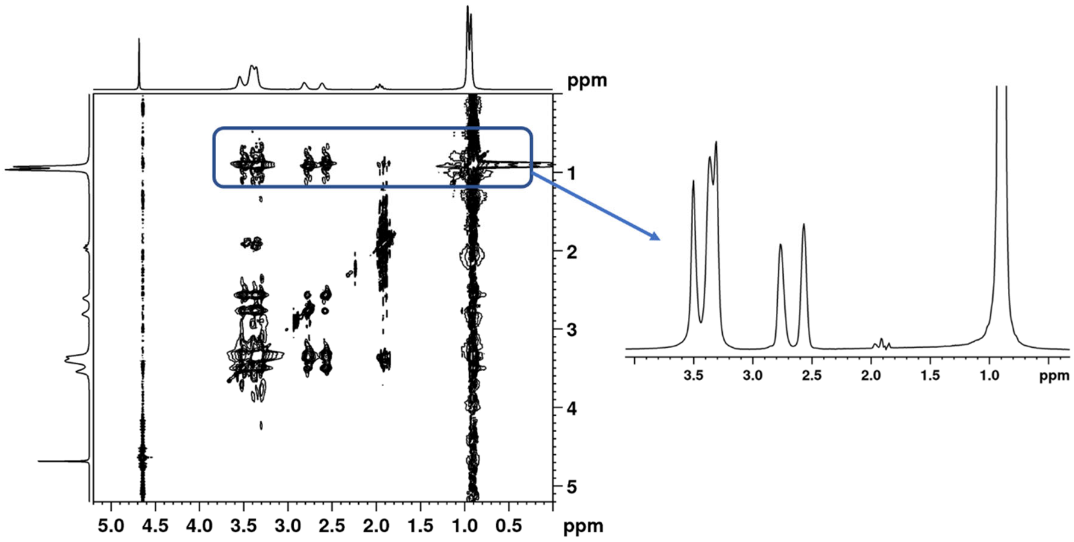

Figure S11. 2D NOESY spectrum of the P(MeOx/IPOx)(14/86) block copolymer in D_2O solution (c = 5 wt%) measured at 295 K with mixing time 600 ms**.** On the right there is 1D slice spectrum extracted from the signal at 1.05 ppm of CH_3 protons of PIPOx units of the NOESY spectrum.

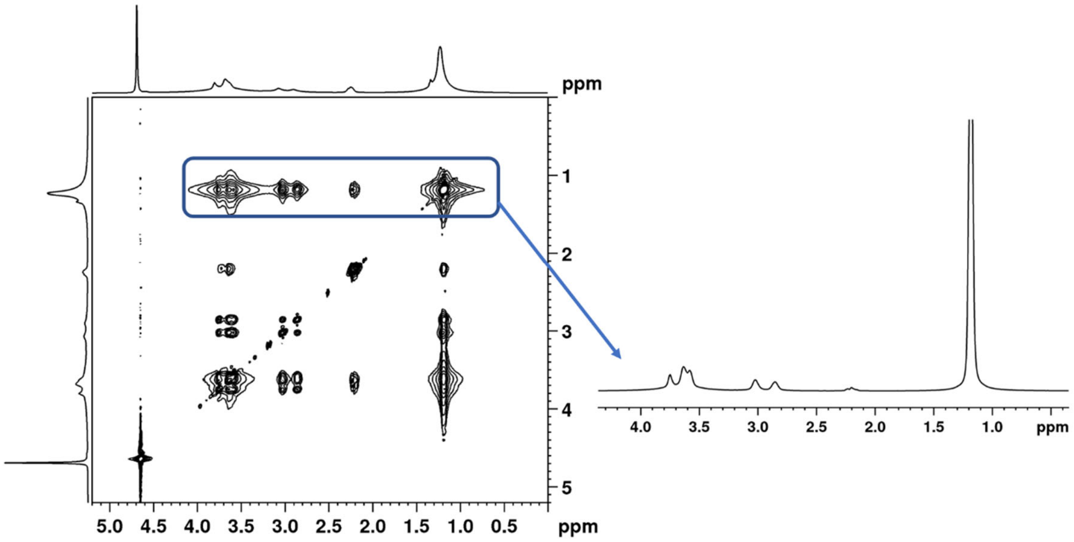

Figure S12. 2D NOESY spectrum of the P(MeOx/IPOx)(14/86) block copolymer in D_2O solution (c = 5 wt%) measured at 320 K with mixing time 600 ms**.** On the right there is 1D slice spectrum extracted from the signal at 1.05 ppm of CH_3 protons of PIPOx units of the NOESY spectrum.

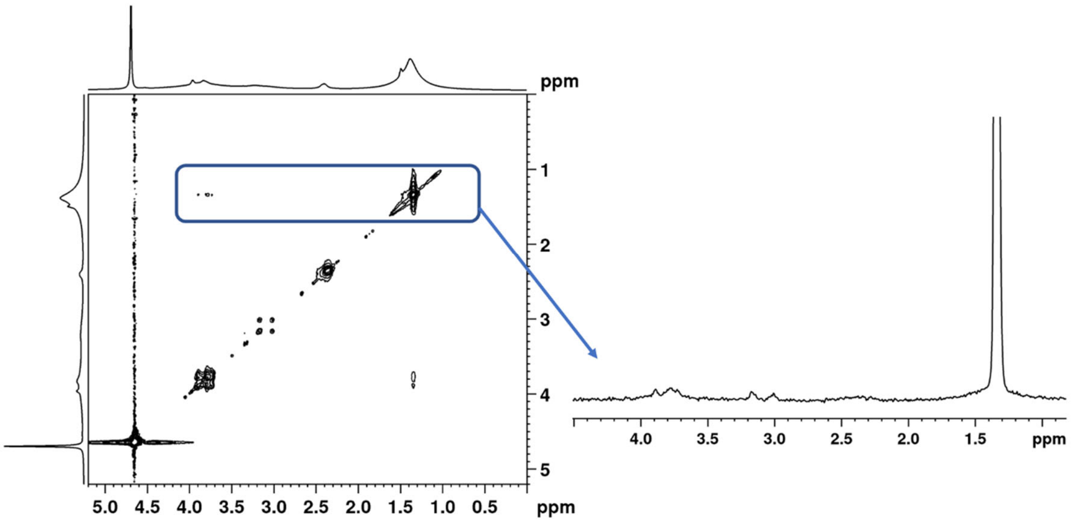

Figure S13. 2D NOESY spectrum of the P(MeOx/IPOx)(14/86) block copolymer in D_2O solution (c = 5 wt%) measured at 335 K with mixing time 600 ms**.** On the right there is 1D slice spectrum extracted from the signal at 1.05 ppm of CH_3 protons of PIPOx units of the NOESY spectrum.

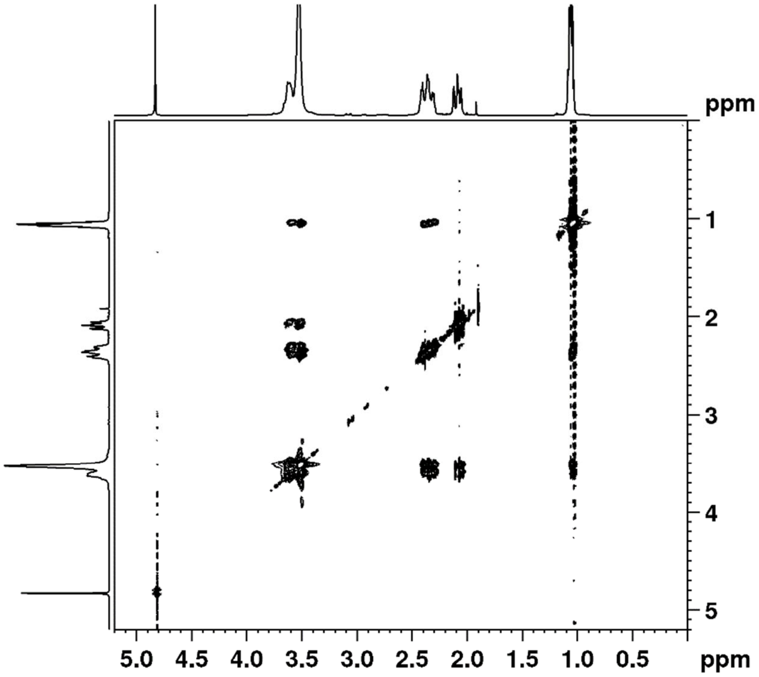

Figure S14. 2D NOESY spectrum of the P(MeOx/EtOx)(28/72) block copolymer in D_2O solution (c = 5 wt%) measured at 295 K with mixing time 600 ms**.**

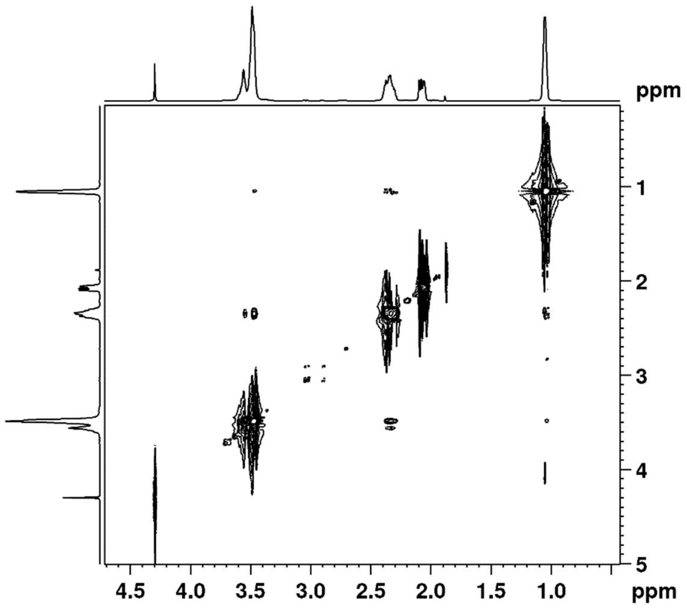

Figure S15. 2D NOESY spectrum of the P(MeOx/EtOx)(28/72) block copolymer in D_2O solution (c = 5 wt%) measured at 340 K with mixing time 600 ms**.**

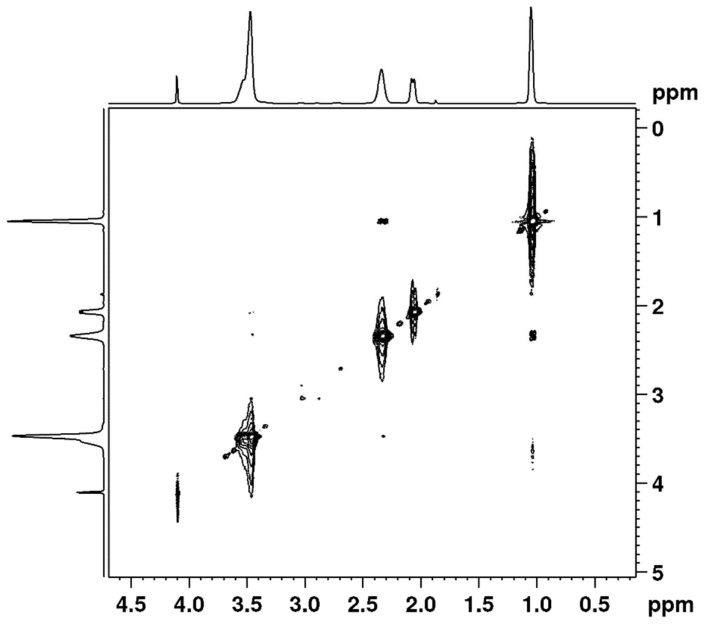

Figure S16. 2D NOESY spectrum of the P(MeOx/EtOx)(28/72) block copolymer in D_2O solution (c = 5 wt%) measured at 360 K with mixing time 600 ms**.**
